# Nomogram Prediction Model for Diabetic Retinopathy Development in Type 2 Diabetes Mellitus Patients: A Retrospective Cohort Study

**DOI:** 10.1155/2021/3825155

**Published:** 2021-09-14

**Authors:** Xiaomei Chen, Qiying Xie, Xiaoxue Zhang, Qi Lv, Xin Liu, Huiying Rao

**Affiliations:** ^1^Department of Ophthalmology, Fujian Provincial Hospital North Branch, Fujian Provincial Geriatric Hospital, Fuzhou, China; ^2^Department of Ophthalmology, Fujian Provincial Hospital, Fuzhou, China

## Abstract

**Background:**

This study is aimed at investigating the systemic risk factors of diabetic retinopathy and further establishing a risk prediction model for DR development in T2DM patients.

**Methods:**

This is a retrospective cohort study including 330 type 2 diabetes mellitus (T2DM) patients who were followed up from December 2012 to November 2020. Multivariable cox regression analysis identifying factors associated with the hazard of developing diabetic retinopathy (DR) was used to construct the DR risk prediction model in the form of nomogram.

**Results:**

50.6% of participants (mean age: 58.60 ± 10.55) were female, and mean duration of diabetes was 7.09 ± 5.36 years. After multivariate cox regression, the risk factors for developing DR were age (HR 1.068, 95%Cl 1.021-1.118, *P* = 0.005), diabetes duration (HR 1.094, 95%Cl 1.018-1.177, *P* = 0.015), HbA1c (HR 1.411, 95%Cl 1.113-1.788, *P* = 0.004), albuminuria (HR 6.908, 95%Cl 1.794-26.599, *P* = 0.005), and triglyceride (HR 1.554, 95%Cl 1.037-2.330, *P* = 0.033). The AUC values of the nomogram for predicting developing DR at 3-, 4-, and 5-year were 0.854, 0.845, and 0.798.

**Conclusion:**

Combining age, diabetes duration, HbA1c, albuminuria, and triglyceride, the nomogram model is effective for early recognition and intervention of individuals at high risk of DR development.

## 1. Introduction

Diabetic retinopathy (DR) is known to be one of the most common microvascular complications of type 2 diabetes mellitus (T2DM) [1]. As the leading cause of vision loss among adults, DR results in nearly 4.8% of 37 million cases of blindness globally [2]. Recent studies related to DR prevalence in China have shown that approximately 9.4%–43.1% of patients with diabetes were diagnosed with DR [3]; with about 113.9 million Chinese adults with diabetes [4], this could be translated into extremely high prevalence of DR in China. However, DR is always symptomless before it enters the late-stage [5]. As DR seriously endangering human health as well as economic sustainability of the national health system, screening for DR is increasingly crucial for individuals of saving vision and for society of saving costs associated with visual impairment and blindness [6, 7]. Nevertheless, the massive population of China as well as a high prevalence of diabetes and relatively insufficient number of clinicians highlight a huge challenge confronting with widespread DR screening. Hence, with the burden of DR on health system becoming increasingly heavy, how to solve the DR screening problem in Chinese medical environment becomes more and more important.

As one of the complications of T2DM, DR is an ocular manifestation of systemic microvascular disease, which means that the development of DR is often accompanied by the development of systemic risk factors and the exacerbation of other diabetic complications. Previous studies have indicated that DR is commonly accompanied by various comorbidities including dyslipidemia, chronic kidney disease, hypertension, hyperglycemia, and anemia [8–10]. Consistent with this notion, diverse researches have also demonstrated multiple different systemic risk factors for DR, such as urine albumin-to-creatinine ratio (UACR), low-density lipoprotein (LDL), apolipoprotein, and hemoglobin A1c (HbA1c) [8, 11, 12]. These findings provide evidence that these easily obtained systemic factors may be capable to be used in building an inexpensive, accurate, and convenient DR development prediction model, therefore, assisting DR screening. Nonetheless to our knowledge, such models have not been extensively explored or used for predicting DR development.

Since the burden of a rising population of T2DM will be increasingly unstoppable, especially in China of a remarkable low doctor-to-patient ratio, prevention is intensely required to reduce the occurrence of associated complications like DR. For this reason, our research tended to build a prediction model for DR development using a nomogram approach, based on the systemic risk factors.

## 2. Methods

### 2.1. Study Population

**Figure 1:**
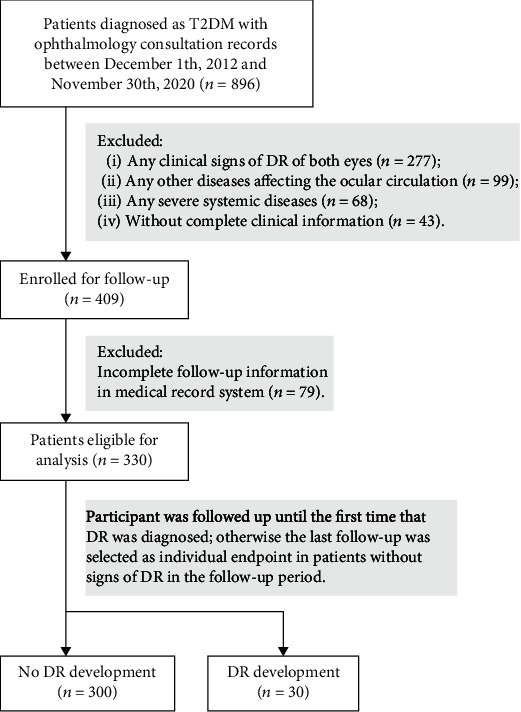
The distribution of study participants. T2DM: type 2 diabetes mellitus; DR: diabetic retinopathy.

This retrospective cohort study was conducted on 330 patients in Fujian Provincial Geriatric Hospital. All medical information in the cohort study was collected from the electronic medical records. Inpatients who were diagnosed as T2DM [13] (ICD-10-CM: E11.900) with contemporaneous ophthalmology consultation records between December 1, 2012, and November 30, 2020, were included in this research. Cases were excluded if the following situations existed at baseline: (1) any clinical signs of DR of both eyes, (2) any other diseases affecting the ocular circulation (e.g., refractive error ≤ −3 diopters, glaucoma, retinal vascular occlusion, and eye trauma), and (3) any severe systemic diseases (e.g., cerebral infarction, myocardial infarction, and history of dialysis). Participant was followed up until the first time that DR was diagnosed; otherwise, the last follow-up was selected as individual endpoint in patients without signs of DR in the follow-up period. The flowchart of Figure 1 demonstrated the distribution of study participants. The research was conducted according to the Declaration of Helsinki and approved by the Research Ethics Committee of Fujian Provincial Geriatric Hospital (registration number: 2020-03-01). Informed written consent was obtained from each study participant.

### 2.2. Data Collection at Baseline

Demographics data (gender, age), medical history (duration of T2DM), physical data (height, weight, and blood pressure), and laboratory parameters were collected at the baseline. The body mass index (BMI) was calculated as weight in kilograms divided by height in meters squared. Blood pressure (BP, mmHg) was measured using a sphygmomanometer after 30 mins of rest. Laboratory parameters included hemoglobin (Hb, g/dL), hematocrit (Hct, %), fasting plasma glucose (FPG, mg/dL), HbA1c (%), serum creatinine (Scr, mg/dL), serum albumin (ALB, g/L), serum total protein (TP, g/L), LDL (mg/dL), triglyceride (TRIG, mg/dL), total cholesterol (TC, mg/dL), and albuminuria (measured qualitatively). Hypertension was defined as systolic blood pressure (SBP) ≥ 140 mmHg, diastolic blood pressure (DBP) ≥ 90 mmHg, antihypertensive drugs usage, or self-reported history of hypertension. Estimated glomerular filtration rate (eGFR) was calculated using the CKD-EPI creatinine equation [14]: 141 × min (Scr/*κ*, 1)^*α*^ × max(Scr/*κ*, 1)^−1.209^ × 0.993^age^ × 1.018 (if female), among which *κ* is 0.9 for males or 0.7 for females, *α* is −0.411 for males or −0.329 for females, max indicates the maximum of 1 or Scr/*κ*, and min indicates the minimum of 1 or Scr/*κ*. All blood and urine samples were collected before 08:00 am.

### 2.3. Evaluation of DR Development at Endpoint

The primary outcome of the cohort endpoint was development of DR. DR assessments were conducted using image evaluation system (2-field fundus photograph) by qualified graders and were reviewed by a retinal professor (H.R.) if two graders held opposing opinions. DR was confirmed if existence of the following retinal lesions [15]: microaneurysms, hard exudates, intraretinal hemorrhagic dots, soft exudates, venous beading, intraretinal microvascular abnormality, neovascularization, preretinal hemorrhage, or vitreous hemorrhage. One eye with the worst retinopathy of the subjects was selected to determine the presence of DR. DR development was defined as DR in any stage at the endpoint of cohort, including nonproliferative diabetic retinopathy and proliferative diabetic retinopathy, while non-DR development was defined as no any signs of DR of both eyes at the endpoint.

### 2.4. Statistical Analysis

Statistical analyses were performed using SPSS software version 26.0 (Chicago, Illinois, USA) and R software version 4.1.0 (The R Foundation for Statistical Computing). All data was tested for normality using Shapiro-Wilk test and histograms. Normally distributed continuous data were demonstrated as mean ± SD, while nonnormally distributed continuous data as (medians, interquartile ranges (IQR)), and categorical data as number and percentage (%). Independent *t*-test was used to compare normally distributed continuous data, while Mann-Whitney *U* test for nonnormally distributed continuous data and chi-square test for categorical data. Cox regressions were conducted using “survival” R package. The outcomes of Cox regressions were expressed as hazard ratios (HRs), confidence interval stated at 95% (95% CI), and *P* value. The nomogram was plotted using “rms” R package, while receiver-operating characteristic (ROC) analyses determining the performance of nomogram to predict DR risk was conducted using “survivalROC” R package. All statistical tests were two-sided with *P* value < 0.05 as statistically significant.

## 3. Results

### 3.1. Participant Characteristics

This retrospective hospital-based cohort study was conducted for a follow-up period with a mean time of 3.66 ± 1.90 years. The mean age of enrolled participants (167 females and 163 males) was 58.60 ± 10.55 years, with the mean duration of T2DM of 7.09 ± 5.36 years.

During the follow-up, 30 participants were found developments into DR (9.1%), while other 300 ones (90.9%) remained non-DR development. The baseline characteristics of these two groups were shown in Table 1. Participants who developed into DR were older at baseline (non-DR development 58.33 years old vs. DR development 61.30 years old) and more likely to be female (non-DR development 51.7% vs. DR development 40.0%), despite not statistically significant. The median (IQR) duration of T2DM for participants developed into DR was 9.5 (8.0) year, significantly longer than that without DR development, which was 6.0 (9.0) years (*P* < 0.01). Patients with DR development were more likely to have hyperglycemia manifested as higher HbA1c and FPG (both *P* < 0.05). In addition, the discrepancy in the incidence of albuminuria appears great between these two groups (*P* < 0.01). Moreover, the level of Scr and TRIG also significantly increased in the groups developed into DR (both *P* < 0.05). Nevertheless, other clinical parameters including BMI, BP, eGFR, TP, ALB, HGB, HCT, TC, LDL, and medication of oral hypoglycemic, insulin, antihypertension, and lipid-lowering remained comparable between these two groups (*P* > 0.05).

### 3.2. DR Development and Risk Factors

To address the risk factors of DR development, we further performed the cox regression analyses between DR development and clinical parameters with three different models (Table 2). Among them, model 1 was a univariate cox regression, model 2 was a multivariate regression with age and sex as covariates, and model 3 was a multivariate regression adjusted by all variables entered into the regression. Model 1 and model 2 demonstrated that age, HbA1c, albuminuria, Scr, ALB, FPG, and TRIG were significantly correlated with DR development (all *P* < 0.05). After controlling for all covariates, our results provided further evidence that the following factors may play essential roles in the development of DR: age (HR 1.068, 95% CI 1.021-1.118, *P* = 0.005), diabetes duration (HR 1.058, 95% CI 1.018-1.177, *P* = 0.015), HbA1c (HR 1.411, 95% CI 1.113-1.788, *P* = 0.004), albuminuria (HR 6.908, 95% CI 1.794-26.599, *P* = 0.005), and TRIG (HR 1.554, 95% CI 1.037-2.330, *P* = 0.033).

### 3.3. Nomogram Model Predicting DR Development

**Figure 2:**
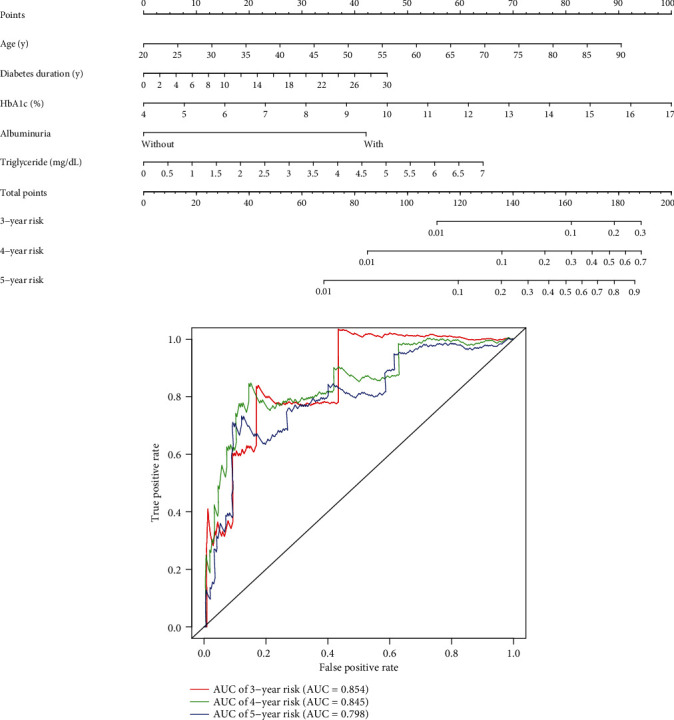
DR prediction model for T2DM patients. (a) Clinical parameter-based nomogram integrating age, diabetes duration, HbA1c, albuminuria, and triglyceride to predict 3-, 4-, and 5-year risks of DR development in patients with T2DM. (b) Time-dependent ROC curves of the nomogram showed AUCs for DR prediction models of 3, 4, and 5 years were 0.854, 0.845, and 0.798, respectively. HbA1c: hemoglobin A1c; AUC: area under the ROC curve; ROC: relative-operating characteristic.

To reach the high ability of DR prediction, the independent risk factors including age, diabetes duration of DR, HbA1c, albuminuria, and triglyceride screened from multivariate cox regression analysis of model 3 were combined to establish a highly accurate developing DR prediction nomogram (Figure 2(a)). The 3-, 4-, and 5-year overall risk of individual DM patients developing DR could be predicted by the nomogram. The great power in predicting DR developing of DM patients was reflected by the time-dependent receiver-operating characteristics (tROC) curve analysis of the nomogram (Figure 2(b)), which showed AUCs for DR prediction models of 3-, 4-, and 5-year were 0.854, 0.845, and 0.798, respectively. Therefore, the systemic factor-based nomogram possibly will help clinicians predict the 3-,4-, and 5-year overall risk of developing DR in patients with DM individually and accurately.

## 4. Discussions

Through this retrospective cohort study, we found several systemic factors including age, duration of diabetes, HbA1c, albuminuria, and TRIG held significant associations with DR development, which remained statistically significant after adjusting for confounding factors. Based on these significant independent systemic variables identified in the multivariate cox regression, we further established a nomogram to formulate a new predictive tool for evaluating risk of DR development after 3-, 4-, and 5-year, which was implied considerable accurate from the AUC analyses.

For the present, Early Treatment Diabetic Retinopathy Study (ETDRS) 7-standard fields color retinal photographs and fundus slit-lamp examination are still the gold standard for DR screening [16, 17]. However, some evident deficiencies are present in the above DR screening modalities: complex of ETDRS photographs and time-consuming of fundus slit-lamp examination, which make them impractical for such large-scale screening in China. Thus, unconventional options are indispensable to circumvent these problems. Some current literatures suggested that the ultrawide-field (UWF) retina imaging providing a single image covering up to 200° of fundus could also be used as a reliable DR screening tool [18]. Data from other studies showed that different quantitative metrics derived from optical coherence tomography (OCT) or optical coherence tomography angiography (OCTA) may be considered as potential discriminant indicators of stage of DR [19]. Nevertheless, the above methods were also hindered by several practical problems, such as cost prohibitive and sophisticated analyses.

Nomogram is considered to be a dependable and practical predictive tool that is capable of generating quantitative probabilities of specific clinical events by incorporating multiple prognostic parameters [20]. The form of nomogram fulfills our desire for a clinically and biologically consolidated model and simultaneously enables our demands for personalized medicine. Through such a form of nomogram, our research established and validated an innovative predictive model for the risk of DR development among individual with T2DM, based on five systemic metrics easy to measure. Recently, investigators have explored the ability of nomogram on DR risk prediction, which were instructive in DR screening: Zhuang et al. built nomogram models to predict the risk of DR and diabetic macular edema (DME) originated from duration of diabetes, urine albumin-to-creatinine ratio (UACR), and LDL [12]; Mo et al. developed an analogous risk nomogram of DR based on other seven systemic predictors [21]. However, researches on the subject have been mostly restricted to limited ability that were only able to predict the current risk of DR but not the future risks. It is been shown that the nomogram based on sex, age, duration of diabetes, and HbA1c could be used to predict NPDR development within 6 months, 1 year, and 3 years in type 1 diabetes mellitus (T1DM) population [22], but the utility of nomogram for predicting DR development in T2DM patients has not been well documented. In this cohort study, through the Cox regressions analyzing the relationships between systemic baseline characteristics and events of DR development at endpoint, we constructed the risk nomogram of DR development after 3, 4, and 5 years in T2DM patients. In addition to indicating the occurrence of DR, this risk prediction model can also guide T2DM patients when to undergo secondary DR screening in the future, thus extending the interval of individual reexamination and alleviating the screening burden.

Sustained hyperglycemia and diabetes duration are widely recognized major risk factors for DR. Our findings demonstrated that HbA1c and diabetes duration were substantially linked with DR development, which was consistent with prior researches [11, 23]. After T2DM is identified, excessively high blood sugar levels produce oxidative stress and microinflammation, which is thought to be a significant pathogenesis of T2DM and associated complications [24, 25]. With duration of the disease increasing and microinflammation developing constantly, the hazard for DR development grows undoubtedly. As a result, adequate blood glucose control, as well as early diagnosis and treatment of DR, is critical.

Albuminuria is a key biochemical biomarker that reflects renal function particularly in diabetic kidney disease (DKD) [26]. Since DR shares comparable etiology with DKD [10, 27], metabolic markers of impaired renal function could not only reflect renal condition but also imply an indirect risk of DR. Multiple prior investigations have shown that albuminuria is a key factor for DR even when other systemic risk factors were controlled [12]. Therefore, albuminuria has also appeared to be highly related to the development of DR in our multivariate cox regression analyses and played an important role in the nomogram model.

Whereas the relationships between lipids and DR were relatively understudied compared to the above indicators, their link has been theorized for many years [8]. In terms of dyslipidemia, our analysis revealed that the development of DR was significantly correlated to TRIG levels. Growing evidences showed that dyslipidemia tends to worsen diabetic retinopathy by inducing inflammation and activating microglia rather than direct lipid extravasation [28] and that aberrant lipid clearance in diabetic retina may play a greater role in oxidative stress and nonenzymatic glycation [29]. Based on our results associated with dyslipidemia and DR, we believe that the clinically accessible and practical TRIG measurement might be a significant indicator for the development of diabetic retinopathy.

Age was shown to be significantly positively associated with the development of DR in our cox regression analyses. This may be explained that age-related alterations in the retinal vasculature expedite the degradation of the retinal perfusion by causing the failure of autoregulation mechanism, which in normal conditions maintains a generally steady blood flow [30]. However, a review of researches revealed that the influence of age on DR remains controversial and varies depending on the populations being investigated. According to the UK Prospective Diabetic Study, older age was found to be a risk factor for DR advancement with a statistically significant relative ratio (RR) of 2.1 [31]. The results of the Wisconsin Epidemiological Study of Diabetic Retinopathy, on the other hand, revealed that older age was a protective factor for diabetic retinopathy [32]. We hypothesize that the confounders in diverse trials, such as variances in environmental, genetic, or lifestyle variables, as well as the type of patients screened, may explain the discrepancies in the influence of age for DR.

During a mean follow-up time of 3.66 ± 1.90 years, 30 (9.09%) of the 330 patients with T2DM included in this cohort eventually developed DR. However, the population included in the study were T2DM patients who had been hospitalized, indicating that they might have a stricter glycemic control regimen. In the real world, however, not all patients with T2DM follow such a rigorous regimen, and some individuals with T2DM may be unaware that they have T2DM until they are in the late stages of DR. This means that DR development may be more severe in the real world. However, even though the nomogram model in this study might be better at predicting DR development in T2DM patients who had an adequate treatment protocol, it still suggests how important DR screening and prevention are in T2DM patients.

To summarize, through the nomogram tool and five systemic factors easily accessible including age, diabetes duration, HbA1c, albuminuria, and triglyceride, we created a reliable prediction model that aided clinicians in the early recognition of individuals at high risk of DR development after 3, 4, and 5 years. Based on this model, clinicians and patients could also implement early medical interventions like as altering treatment scheme to decline the risk of DR. In other words, this quantitative framework was of great significance for disease management of DR in high-risk population, manifested in indicating and delaying the development of DR in T2DM.

There are still a few limitations in this study. Firstly, the lack of external validation is one of the significant limitations of our study. In this context, additional research is required to replicate and externally validate the findings of this study. Secondly, due to the limited sample size, the endpoint event in our cohort study was defined as DR development including both NPDR and PDR. Study's findings would have been more enriched if development of NPDR and PDR had been analyzed separately. Thirdly, because all data in this investigation were collected from a medical recording system or fundus color photography rather than fundus fluorescence angiography imaging, the DR diagnosis might lack strictness. The prediction model in this study may, however, remain generalized, and it is desired that future prospective studies can be carried out to assess the accuracy of this model in the real world and to further enhance it.

## Figures and Tables

**Table 1:** Characteristics of DR nondevelopment group and DR development group.

Characteristics	Non-DR development (*n* = 300)	DR development (*n* = 30)	*P* value
Age (years)	58.33 ± 10.64	61.30 ± 9.31	†0.142
Sex (female)	155 (51.7%)	12 (40.0%)	‡0.223
Diabetes duration (years)	6.00, 9.00	9.50, 8.00	§0.009^∗∗^
BMI (kg/m^2^)	25.62 ± 3.63	25.72 ± 3.55	†0.889
HBP	123 (41.0%)	13 (43.3%)	‡0.804
SBP (mmHg)	127.81 ± 17.28	130.03 ± 18.97	†0.505
DBP (mmHg)	76.03 ± 11.96	75.9 ± 11.08	†0.953
HbA1c (%)	7.23 ± 1.55	8.38 ± 2.26	†0.010^∗^
Albuminuria (+~+++)	11 (3.7%)	5 (16.7%)	‡0.002^∗∗^
Scr (mg/dL)	84.89 ± 23.84	103.49 ± 43.64	†0.028^∗^
eGFR (mL/min/1.73 m^2^)	79.11 ± 21.27	71.82 ± 29.60	†0.197
TP (g/L)	70.74 ± 6.08	70.3 ± 8.88	†0.794
ALB (g/L)	44.29 ± 4.17	42.8 ± 5.47	†0.071
HGB (g/dL)	134.12 ± 15.51	137.57 ± 19.7	†0.259
HCT (%)	39.05 ± 4.23	39.58 ± 5.75	†0.523
FPG (mg/dL)	6.89, 2.55	7.81, 5.82	§0.023^∗^
TC (mg/dL)	4.27 ± 0.89	4.05 ± 1.01	†0.204
LDL (mg/dL)	2.50 ± 0.75	2.34 ± 0.74	†0.260
TRIG (mg/dL)	1.31, 1.00	1.89, 1.32	§0.011^∗^
Oral hypoglycemic	265 (88.3%)	28 (93.3%)	‡0.408
Insulin (unit)	15.00, 27.00	16.50, 20.00	§0.743
Antihypertension	94 (31.3%)	10 (33.3%)	‡0.822
Lipid-lowering	224 (74.7%)	21 (70.0%)	‡0.577

Results are expressed as mean ± SD, percentages, or as medians, IQR; *P* values were compared by independent *t*-test, Mann-Whitney *U* test or *χ*^2^ test as appropriate. ^∗^*P* < 0.05, ^∗∗^*P* < 0.01.†Values for comparisons between groups by independent samples *t*-test. ‡Values for comparisons between groups by *χ*^2^ test. §Values for comparisons between groups by Mann-Whitney *U* test. DR: diabetic retinopathy; BMI: body mass index; HBP: hypertension; SBP: systolic blood pressure; DBP: diastolic blood pressure; HbA1c: hemoglobin A1c; Scr: serum creatinine; eGFR: estimated glomerular filtration rate; TP: total protein; ALB: serum albumin; HGB: hemoglobin; HCT: hematocrit; FPG: fasting plasma glucose; TC: total cholesterol; LDL: low-density lipoprotein; TRIG: triglyceride.

**Table 2:** Cox regression for DR development with clinical characteristics. †Model 1: univariate cox regression. ‡Model 2: age and sex were adjusted by each other; all other variables were adjusted by age and sex. §Model 3: all variables were entered into this multivariate regression analysis model. The outcomes of Cox regressions were expressed as HRs, 95% CI, and *P* value. ^∗^*P* < 0.05, ^∗∗^*P* < 0.01. DR: diabetic retinopathy; BMI: body mass index; SBP: systolic blood pressure; DBP: diastolic blood pressure; HbA1c: hemoglobin A1c; Scr: serum creatinine; eGFR: estimated glomerular filtration rate; TP: total protein; ALB: serum albumin; HGB: hemoglobin; HCT: hematocrit; FPG: fasting plasma glucose; TC: total cholesterol; LDL: low-density lipoprotein; TRIG, triglyceride.

Characteristics	†Model 1		Model 2		Model 3	
	HR (95%Cl)	*P* value	HR (95%Cl)	*P* value	HR (95%Cl)	*P* value
Age (years)	1.036 (0.997−1.077)	0.067	1.041 (1.001−1.082)	0.043^∗^	1.068 (1.021−1.118)	0.005^∗∗^
Sex (female)	0.601 (0.289−1.249)	0.173	0.544 (0.260−1.139)	0.107	1.058 (0.349−3.209)	0.920
Diabetes duration (years)	1.058 (0.993−1.127)	0.080	1.046 (0.982−1.114)	0.163	1.094 (1.018−1.177)	0.015^∗^
BMI (kg/m^2^)	0.992 (0.893−1.103)	0.886	1.005 (0.897−1.126)	0.929	0.988 (0.867−1.126)	0.856
SBP (mmHg)	1.008 (0.988−1.029)	0.429	1.006 (0.986−1.025)	0.572	1.007 (0.984−1.031)	0.564
HbA1c (%)	1.297 (1.129−1.489)	<0.001^∗∗^	1.406 (1.206−1.640)	<0.001^∗∗^	1.411 (1.113−1.788)	0.004^∗∗^
Albuminuria (+~+++)	4.804 (1.789−12.895)	0.002^∗∗^	4.952 (1.826−13.430)	0.002^∗∗^	6.908 (1.794−26.599)	0.005^∗∗^
Scr (mg/dL)	1.013 (1.005−1.021)	0.002^∗∗^	1.011 (1.001−1.020)	0.022^∗^	1.003 (0.990−1.016)	0.634
TP (g/L)	0.969 (0.917−1.024)	0.263	0.967 (0.915−1.022)	0.232	1.000 (0.926−1.080)	0.993
ALB (g/L)	0.889 (0.821−0.962)	0.004^∗∗^	0.875 (0.805−0.952)	0.002^∗∗^	0.895 (0.768−1.044)	0.157
HGB (g/dL)	1.012 (0.990−1.034)	0.281	1.010 (0.984−1.037)	0.445	1.067 (0.965−1.179)	0.205
HCT (%)	1.038 (0.958−1.125)	0.358	1.022 (0.929−1.123)	0.658	0.890 (0.625−1.268)	0.520
FPG (mg/dL)	1.126 (1.047−1.211)	0.001^∗∗^	1.133 (1.058−1.213)	<0.001^∗∗^	1.038 (0.931−1.158)	0.500
TC (mg/dL)	0.796 (0.532−1.191)	0.267	0.903 (0.591−1.380)	0.637	0.738 (0.327−1.668)	0.465
LDL (mg/dL)	0.725 (0.450−1.169)	0.187	0.818 (0.501−1.338)	0.424	0.727 (0.296−1.787)	0.487
TRIG (mg/dL)	1.371 (1.058−1.778)	0.017^∗^	1.488 (1.125−1.969)	0.005^∗∗^	1.554 (1.037−2.330)	0.033^∗^

## Data Availability

The data related to this article can be publicly available after the article accepted.
